# The iron phosphate CaFe_3_(PO_4_)_3_O

**DOI:** 10.1107/S160053680902827X

**Published:** 2009-07-22

**Authors:** Mourad Hidouri, Mongi Ben Amara

**Affiliations:** a Faculté des Sciences de Monastir, 5019, Monastir, Tunisia.

## Abstract

A new iron phosphate, calcium triiron(III) tris­(phosphate) oxide, CaFe_3_(PO_4_)_3_O, has been isolated and shown to exhibit a three-dimensional structure built by FeO_6_ octa­hedra, FeO_5_ trigonal bipyramids and PO_4_ tetra­hedra. The FeO_*x*_ (*x* = 5, 6) polyhedra are linked through common corners and edges, forming [Fe_6_O_28_]_∞_ chains with branches running along [010]. Adjacent chains are connected by the phosphate groups *via* common corners and edges, giving rise to a three-dimensional framework analogous to those of the previously reported SrFe_3_(PO_4_)_3_O and Bi_0.4_Fe_3_(PO_4_)_3_O structures, in which the Ca^2+^ cations occupy a single symmetry non-equivalent cavity.

## Related literature

The inter­est in iron phosphates has increased following the discovery of LiFePO_4_ with olivine-type structure, which is the most promising electrode material for Li-ion batteries, see: Padhi *et al.* (1997 ▶). The title compound is isostructural to the iron phosphates Bi_0.4_Fe_3_(PO_4_)_3_ (Benabad *et al.*, 2000 ▶) and SrFe_3_(PO_4_)_3_O (Morozov *et al.*, 2003 ▶). For ionic radii, see: Shannon (1976 ▶). For P—O distances in orthophosphate groups, see: Baur (1974 ▶).  For Ca—O distances in heptacoordinated Ca^2+^ ions in Ca_3_(PO_4_)_2_, see: Mathew *et al.* (1977 ▶). For Fe—O distances for five-coordinated Fe^3+^ ions in  NaCaFe_3_(PO_4_)_4_, see: Hidouri *et al.* (2003 ▶).The valences of the cations were calculated using the Brown & Altermatt (1985 ▶) method.

## Experimental

### 

#### Crystal data

CaFe_3_(PO_4_)_3_O
*M* *_r_* = 508.54
Monoclinic, 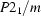

*a* = 7.521 (2) Å
*b* = 6.330 (2) Å
*c* = 10.160 (2) Å
β = 100.03 (2)°
*V* = 476.3 (2) Å^3^
*Z* = 2
Mo *K*α radiation
μ = 5.63 mm^−1^
*T* = 293 K
0.36 × 0.22 × 0.22 mm

#### Data collection

Enraf–Nonius TurboCAD-4 diffractometer
Absorption correction: ψ scan (North *et al.*, 1968 ▶) *T*_min_ = 0.193, *T*_max_ = 0.293
2072 measured reflections
1493 independent reflections
1412 reflections with *I* > 2σ(*I*)
*R*_int_ = 0.035
2 standard reflections frequency: 120 min intensity decay: 6.0%

#### Refinement

*R*[*F* ^2^ > 2σ(*F* ^2^)] = 0.031
*wR*(*F* ^2^) = 0.088
*S* = 1.12
1493 reflections
113 parameters
Δρ_max_ = 0.63 e Å^−3^
Δρ_min_ = −1.59 e Å^−3^

### 

Data collection: *CAD-4 EXPRESS* (Enraf–Nonius, 1994 ▶); cell refinement: *CAD-4 EXPRESS*; data reduction: *XCAD4* (Harms & Wocadlo, 1995 ▶); program(s) used to solve structure: *SHELXS97* (Sheldrick, 2008 ▶); program(s) used to refine structure: *SHELXL97* (Sheldrick, 2008 ▶); molecular graphics: *DIAMOND* (Brandenburg, 1998 ▶); software used to prepare material for publication: *WinGX* (Farrugia, 1999 ▶).

## Supplementary Material

Crystal structure: contains datablocks I, global. DOI: 10.1107/S160053680902827X/er2067sup1.cif

Structure factors: contains datablocks I. DOI: 10.1107/S160053680902827X/er2067Isup2.hkl

Additional supplementary materials:  crystallographic information; 3D view; checkCIF report

## Figures and Tables

**Fig. 1.**
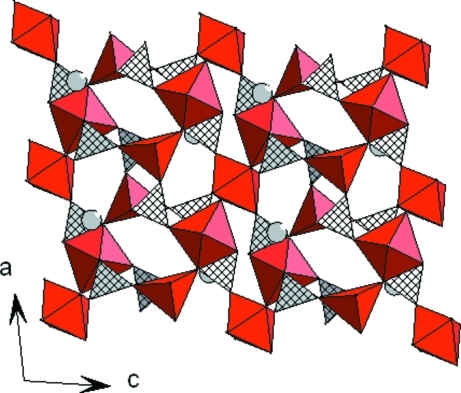
: The CaFe3(PO4)3O structure as projected along the [010] direction.

**Fig. 2.**
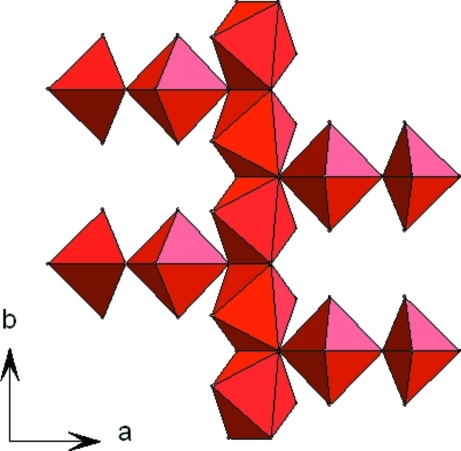
: A view of the [Fe6O28]∞ chain running along the [010] direction.

**Fig. 3.**
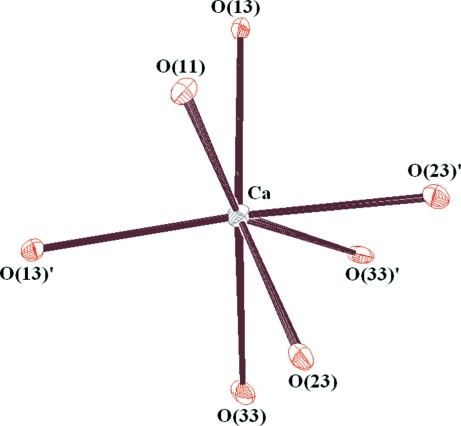
: The environment of the Ca2+ cations showing the anisotropic atomic displacements.

## supplementary crystallographic information

### Comment

Iron phosphates are extensively studied for their rich structural chemistry owing to the possible occurrence of both +2 and +3 oxidation states for iron and the tendecy of its coordination polyhedra to form with the phosphate groups a variety of frameworks. Such adaptative crystal chemistry provides new and exciting aventures in the exploration of the intrinsic relationship between structure and composition. The interest in these materials is further accentuated since the discovery of LiFePO_4_ with olivine-type structure the most promising electrode material for Li-ion batteries (Padhi *et al.*, 1997).

As a part of a systematic exploration of the A_2_O—MO—Fe_2_O_3_—P_2_O_5_ (A = alkali metal, *M* = divalent cation) in a search of new iron phosphates with interesting structures and subsequently intriguing properties, we describe here the structure of CaFe_3_(PO_4_)_3_O, extracted from a mixture of nominal composition LiCaFe_3_(PO_4_)_4_. This compound is isostructural to the previously reported iron phosphates Bi_0.4_Fe_3_(PO_4_)_3_ (Benabad *et al.*, 2000) and SrFe_3_(PO_4_)_3_O (Morozov *et al.*, 2003). Its structure, shown in figure 1, is built from a three-dimensional arrangement based on two crystallographically distinct FeO_6_ octahedra, one symmetry non equivalent FeO_5_ polyhedron and three symmetry distinct PO_4_ tetrahedra. The Fe polyhedra form [Fe_6_O_28_]_∞_ chains with branches running along the [010] direction. In such chains (Fig. 2), each Fe(1)O_6_ octahedron shares two opposite edges with two equivalent octahedra, one of the equatorial oxo-ligands forming each of the common edges being also shared with one Fe(2)O_6_ octahedron. The latter is corner-linked with one one Fe(2)O_5_ polyhedron to form the branches of the chain. The conntection of these chains is ensured by the phosphate tetrahedra in such a way that each PO_4_ connects two adjacent chains either by sharing one edge with one chain and one corner with the other (P(1)O_4_) or by sharing three corners with a same chain and the fourth with the other (P(2)O_4_ and P(3)O_4_). The three-dimensional framework constructed in this way delimits a single symmetry non equivalent cavity occupied by the Ca^2+^ cations.

The FeO_6_ octahedra are both highly distorted as indicated by the Fe—O distances ranging from 1.986 (2) to 2.114 (2) Å for Fe(1)O_6_ and from 1.870 (2) to 2.183 (3) Å for Fe(2)O_6_ with average values of 2.037 (2) Å and 2.019 (3) Å, respectively, close to that 2.03 Å, predicted by Shannon for octahedral Fe^3+^ ions (Shannon, 1976). The FeO_5_ polyhedron is also very distorted with Fe—O distances ranging from 1.872 (4) to 1.986 (2) Å. The mean distance of 1.940 (4) Å is consistent with those 1.946 Å and 1.956 Å, observed for five-coordinated Fe^3+^ ions in NaCaFe_3_(PO_4_)_4_ (Hidouri *et al.*,  2003). The PO_4_ tetrahedra have P—O distances in the range 1.513 (3)–1.561 (3) Å with an overall distance of 1.535 (3) Å, close to that 1.537 calculated for the monophosphate groups (Baur, 1974). The Ca^2+^ cations occupy a single non equivalent site delimited by the Fe/P/O network. Its environement (Fig.3) is consisted by seven oxygen atoms with four Ca—O distances included between 2.391 (3) and 2.514 (2) Å showing the CaO_7_ polyhedron to be highly distorted. The mean Ca—O distance of 2.462 (2) Å is in the range of those previously reported for heptacoordinated Ca^2+^ ions in Ca_3_(PO_4_)_2_ (Mathew *et al.*, 1977). The valences of all the cations were calculated using the Brown & Altermatt method (Brown & Altermatt, 1985). The calculated values of 1.85, 2.86, 3.10, 3.09, 4.94, 5.03 and 5.02 for Ca, Fe(1), Fe(2), Fe(3), P(1), P(2) and P(3), respectively are consistent with their respective oxidation numbers of 2.0, 3.0, 3.0, 3.0, 5.0, 5.0 and 5.0.

The structural similarity between the title compound and the iron phosphates SrFe_3_(PO_4_)_3_O and Bi_0.4_Fe_3_(PO_4_)_3_O shows the great flexibility of the [Fe_3_P_3_O_13_]_∞_ framework which seems to accomodate various cations. Further invstigation of the chemical stablity of this structural type by including other cations would be of interest.

### Experimental

Single crystals of the title compound were isolated during an attempt to crystallize LiCaFe_3_(PO_4_)_4_ in a flux of lithium dimolybdate Li_2_Mo_2_O_7_ in an atomic ratio, P: Mo = 8:1. Appropriate amounts of LiNO_3_, CaCO_3_, Fe(NO_3_)_3_.9H_2_O, (NH_4_)_2_HPO_4_ and MoO_3_ were firstly dissolved in nitric acid and the solution obtained was dried for 24 h at 353 K. After grinding in an agate mortar to ensure its best homogeneity, the dry residue was heated in a platinum crucible to 673 K for 24 h in order to remove the decomposition products: NO_2_, NH_3_ and H_2_O. The sample was then reground, melted at 1173 K for 1 h and subsequently cooled at a 10 °.h^-1^ rate to 673 K after which the furnace was turned off. The final product was washed with warm water in order to dissolve the flux. From the mixture, dark brown and irregularely shaped crystals of CaFe_3_(PO_4_)_3_O were extracted.

### Refinement

The Fe and Ca atoms were loctaed by direct methods and the remaining atoms were found by successive difference Fourier maps. All atomic positions were refined with anisotrop displacement parameterers.

### Crystal data

**Table:**

CaFe_3_(PO_4_)_3_O	*F*(000) = 494
*M**_r_* = 508.54	*D*_x_ = 3.546 Mg m^−^^3^
Monoclinic, *P*2_1_/*m*	Mo *K*α radiation, λ = 0.71073 Å
Hall symbol: -P 2yb	Cell parameters from 25 reflections
*a* = 7.521 (2) Å	θ = 8.9–12.5°
*b* = 6.330 (2) Å	µ = 5.63 mm^−^^1^
*c* = 10.160 (2) Å	*T* = 293 K
β = 100.03 (2)°	Prism, brown
*V* = 476.3 (2) Å^3^	0.36 × 0.22 × 0.22 mm
*Z* = 2	

### Data collection

**Table:**

Enraf–Nonius TurboCAD-4 diffractometer	1412 reflections with *I* > 2σ(*I*)
Radiation source: fine-focus sealed tube	*R*_int_ = 0.035
graphite	θ_max_ = 29.9°, θ_min_ = 2.0°
ω/2θ scans	*h* = −1→10
Absorption correction: ψ scan (North *et al.*, 1968)	*k* = −1→8
*T*_min_ = 0.193, *T*_max_ = 0.293	*l* = −14→14
2072 measured reflections	2 standard reflections every 120 min
1493 independent reflections	intensity decay: 6.0%

### Refinement

**Table:**

Refinement on *F*^2^	Primary atom site location: structure-invariant direct methods
Least-squares matrix: full	Secondary atom site location: difference Fourier map
*R*[*F*^2^ > 2σ(*F*^2^)] = 0.031	*w* = 1/[σ^2^(*F*_o_^2^) + (0.0585*P*)^2^ + 0.6592*P*] where *P* = (*F*_o_^2^ + 2*F*_c_^2^)/3
*wR*(*F*^2^) = 0.088	(Δ/σ)_max_ < 0.001
*S* = 1.12	Δρ_max_ = 0.63 e Å^−^^3^
1493 reflections	Δρ_min_ = −1.59 e Å^−^^3^
113 parameters	Extinction correction: *SHELXL97* (Sheldrick, 2008), Fc^*^=kFc[1+0.001xFc^2^λ^3^/sin(2θ)]^-1/4^
0 restraints	Extinction coefficient: 0.173 (7)

### Special details

**Table:**

Geometry. All e.s.d.'s (except the e.s.d. in the dihedral angle between two l.s. planes) are estimated using the full covariance matrix. The cell e.s.d.'s are taken into account individually in the estimation of e.s.d.'s in distances, angles and torsion angles; correlations between e.s.d.'s in cell parameters are only used when they are defined by crystal symmetry. An approximate (isotropic) treatment of cell e.s.d.'s is used for estimating e.s.d.'s involving l.s. planes.
Refinement. Refinement of *F*^2^ against ALL reflections. The weighted *R*-factor *wR* and goodness of fit *S* are based on *F*^2^, conventional *R*-factors *R* are based on *F*, with *F* set to zero for negative *F*^2^. The threshold expression of *F*^2^ > σ(*F*^2^) is used only for calculating *R*-factors(gt) *etc*. and is not relevant to the choice of reflections for refinement. *R*-factors based on *F*^2^ are statistically about twice as large as those based on *F*, and *R*- factors based on ALL data will be even larger.

### Fractional atomic coordinates and isotropic or equivalent isotropic displacement parameters (Å^2^)

**Table:**

	*x*	*y*	*z*	*U*_iso_*/*U*_eq_	
Ca	0.66161 (10)	−0.2500	0.19595 (7)	0.00891 (18)	
Fe1	0.0000	−0.5000	0.0000	0.00627 (16)	
Fe2	−0.64926 (7)	−0.7500	0.20179 (5)	0.00561 (16)	
Fe3	−0.21388 (7)	−0.7500	0.43643 (5)	0.00684 (16)	
P1	−0.31703 (11)	−0.7500	0.11247 (8)	0.0052 (2)	
O11	−0.5087 (3)	−0.7500	0.0310 (2)	0.0081 (5)	
O12	−0.3598 (3)	−0.7500	0.2566 (3)	0.0080 (5)	
O13	−0.2107 (2)	−0.5489 (3)	0.09386 (18)	0.0083 (3)	
P2	0.26341 (12)	−0.2500	0.23940 (9)	0.0054 (2)	
O21	0.0855 (3)	−0.2500	0.1340 (3)	0.0084 (5)	
O22	0.2123 (4)	−0.2500	0.3770 (3)	0.0130 (5)	
O23	0.3790 (2)	−0.4390 (3)	0.21363 (18)	0.0091 (3)	
P3	0.21762 (12)	−0.7500	0.48890 (9)	0.0064 (2)	
O31	0.0256 (4)	−0.7500	0.4084 (3)	0.0140 (5)	
O32	0.3513 (4)	−0.7500	0.3933 (3)	0.0119 (5)	
O33	−0.2479 (3)	−1.0599 (3)	0.41460 (18)	0.0116 (4)	
O	−0.8765 (3)	−0.7500	0.0924 (2)	0.0070 (5)	

### Atomic displacement parameters (Å^2^)

**Table:**

	*U*^11^	*U*^22^	*U*^33^	*U*^12^	*U*^13^	*U*^23^
Ca	0.0108 (3)	0.0090 (3)	0.0072 (3)	0.000	0.0024 (2)	0.000
Fe1	0.0075 (2)	0.0054 (2)	0.0068 (3)	−0.00023 (16)	0.00379 (17)	0.00011 (17)
Fe2	0.0066 (3)	0.0050 (3)	0.0057 (2)	0.000	0.00243 (17)	0.000
Fe3	0.0111 (3)	0.0060 (3)	0.0036 (3)	0.000	0.00167 (18)	0.000
P1	0.0065 (4)	0.0052 (4)	0.0045 (4)	0.000	0.0028 (3)	0.000
O11	0.0075 (11)	0.0106 (12)	0.0066 (11)	0.000	0.0019 (9)	0.000
O12	0.0096 (11)	0.0097 (12)	0.0058 (10)	0.000	0.0042 (8)	0.000
O13	0.0089 (7)	0.0075 (8)	0.0096 (8)	−0.0013 (6)	0.0048 (6)	0.0000 (6)
P2	0.0077 (4)	0.0049 (4)	0.0038 (4)	0.000	0.0019 (3)	0.000
O21	0.0099 (11)	0.0089 (11)	0.0065 (10)	0.000	0.0011 (9)	0.000
O22	0.0189 (13)	0.0164 (13)	0.0047 (11)	0.000	0.0050 (9)	0.000
O23	0.0106 (8)	0.0055 (7)	0.0114 (8)	0.0007 (6)	0.0028 (6)	−0.0005 (6)
P3	0.0103 (4)	0.0053 (4)	0.0044 (4)	0.000	0.0032 (3)	0.000
O31	0.0114 (12)	0.0208 (14)	0.0093 (12)	0.000	0.0005 (9)	0.000
O32	0.0135 (12)	0.0167 (13)	0.0070 (11)	0.000	0.0057 (9)	0.000
O33	0.0213 (9)	0.0059 (7)	0.0078 (8)	−0.0006 (7)	0.0031 (7)	0.0016 (6)
O	0.0065 (10)	0.0070 (11)	0.0068 (10)	0.000	−0.0005 (8)	0.000

### Geometric parameters (Å, °)

**Table:**

Ca—O11^i^	2.391 (3)	Fe3—O33	1.986 (2)
Ca—O13^ii^	2.434 (2)	Fe3—O33^xii^	1.986 (2)
Ca—O13^iii^	2.434 (2)	P1—O13	1.532 (2)
Ca—O23	2.473 (3)	P1—O13^xii^	1.532 (2)
Ca—O23^iv^	2.473 (3)	P1—O11	1.532 (3)
Ca—O33^v^	2.514 (2)	P1—O12	1.554 (3)
Ca—O33^vi^	2.514 (2)	P1—Ca^ix^	3.2878 (10)
Ca—P2	3.102 (4)	P1—Ca^xiii^	3.2878 (10)
Ca—P3^vii^	3.1724 (16)	O11—Ca^i^	2.391 (3)
Ca—P1^ii^	3.2878 (10)	O13—Ca^ix^	2.434 (2)
Ca—P1^v^	3.2878 (10)	P2—O22	1.513 (3)
Fe1—O^viii^	1.9860 (17)	P2—O23^iv^	1.528 (2)
Fe1—O^ii^	1.9860 (17)	P2—O23	1.528 (2)
Fe1—O13	2.011 (2)	P2—O21	1.561 (3)
Fe1—O13^i^	2.011 (2)	O21—Fe1^xiv^	2.1135 (18)
Fe1—O21^i^	2.1135 (18)	O22—Fe3^xi^	1.894 (3)
Fe1—O21	2.1135 (18)	O23—Fe2^ii^	1.981 (2)
Fe2—O	1.870 (3)	P3—O32	1.515 (3)
Fe2—O32^ix^	1.945 (3)	P3—O31	1.530 (3)
Fe2—O23^ix^	1.981 (2)	P3—O33^xv^	1.544 (2)
Fe2—O23^x^	1.981 (2)	P3—O33^xvi^	1.544 (2)
Fe2—O12	2.151 (4)	P3—Ca^vii^	3.1724 (16)
Fe2—O11	2.183 (3)	O32—Fe2^ii^	1.945 (3)
Fe2—P1	2.803 (3)	O33—P3^xvi^	1.544 (2)
Fe3—O31	1.872 (4)	O33—Ca^xiii^	2.514 (2)
Fe3—O22^xi^	1.894 (3)	O—Fe1^xvii^	1.9860 (17)
Fe3—O12	1.960 (3)	O—Fe1^ix^	1.9860 (17)
			
O11^i^—Ca—O13^ii^	75.42 (7)	O—Fe2—P1	125.58 (10)
O11^i^—Ca—O13^iii^	75.42 (7)	O32^ix^—Fe2—P1	118.50 (10)
O13^ii^—Ca—O13^iii^	102.03 (10)	O23^ix^—Fe2—P1	85.83 (5)
O11^i^—Ca—O23	78.17 (9)	O23^x^—Fe2—P1	85.83 (5)
O13^ii^—Ca—O23	93.59 (8)	O31—Fe3—O22^xi^	108.28 (14)
O13^iii^—Ca—O23	144.58 (7)	O31—Fe3—O12	104.83 (13)
O11^i^—Ca—O23^iv^	78.17 (9)	O22^xi^—Fe3—O12	146.89 (12)
O13^ii^—Ca—O23^iv^	144.58 (7)	O31—Fe3—O33	95.26 (6)
O13^iii^—Ca—O23^iv^	93.59 (8)	O22^xi^—Fe3—O33	95.17 (6)
O23—Ca—O23^iv^	57.88 (11)	O12—Fe3—O33	81.70 (6)
O11^i^—Ca—O33^v^	149.65 (5)	O31—Fe3—O33^xii^	95.26 (6)
O13^ii^—Ca—O33^v^	133.04 (8)	O22^xi^—Fe3—O33^xii^	95.17 (6)
O13^iii^—Ca—O33^v^	86.46 (7)	O12—Fe3—O33^xii^	81.70 (6)
O23—Ca—O33^v^	105.74 (8)	O33—Fe3—O33^xii^	162.15 (12)
O23^iv^—Ca—O33^v^	78.93 (9)	O13—P1—O13^xii^	112.33 (16)
O11^i^—Ca—O33^vi^	149.65 (5)	O13—P1—O11	113.29 (9)
O13^ii^—Ca—O33^vi^	86.46 (7)	O13^xii^—P1—O11	113.29 (9)
O13^iii^—Ca—O33^vi^	133.04 (8)	O13—P1—O12	108.34 (9)
O23—Ca—O33^vi^	78.93 (9)	O13^xii^—P1—O12	108.34 (9)
O23^iv^—Ca—O33^vi^	105.74 (8)	O11—P1—O12	100.34 (15)
O33^v^—Ca—O33^vi^	57.20 (9)	O13—P1—Fe2	123.62 (8)
O11^i^—Ca—P2	79.81 (9)	O13^xii^—P1—Fe2	123.62 (8)
O13^ii^—Ca—P2	121.53 (5)	O11—P1—Fe2	50.73 (11)
O13^iii^—Ca—P2	121.53 (5)	O12—P1—Fe2	49.61 (11)
O33^v^—Ca—P2	89.64 (8)	O13—P1—Ca^ix^	44.13 (8)
O33^vi^—Ca—P2	89.64 (8)	O13^xii^—P1—Ca^ix^	151.82 (9)
O11^i^—Ca—P3^vii^	168.11 (7)	O11—P1—Ca^ix^	93.19 (4)
O13^ii^—Ca—P3^vii^	111.42 (6)	O12—P1—Ca^ix^	74.294 (19)
O13^iii^—Ca—P3^vii^	111.42 (6)	Fe2—P1—Ca^ix^	80.22 (2)
O23—Ca—P3^vii^	91.45 (7)	O13—P1—Ca^xiii^	151.82 (9)
O23^iv^—Ca—P3^vii^	91.45 (7)	O13^xii^—P1—Ca^xiii^	44.13 (8)
P2—Ca—P3^vii^	88.29 (7)	O11—P1—Ca^xiii^	93.19 (4)
O11^i^—Ca—P1^ii^	77.77 (2)	O12—P1—Ca^xiii^	74.294 (19)
O13^ii^—Ca—P1^ii^	25.99 (5)	Fe2—P1—Ca^xiii^	80.22 (2)
O13^iii^—Ca—P1^ii^	126.71 (6)	Ca^ix^—P1—Ca^xiii^	148.58 (4)
O23—Ca—P1^ii^	68.54 (6)	P1—O11—Fe2	96.36 (14)
O23^iv^—Ca—P1^ii^	124.46 (6)	P1—O11—Ca^i^	140.38 (15)
O33^v^—Ca—P1^ii^	132.14 (5)	Fe2—O11—Ca^i^	123.26 (13)
O33^vi^—Ca—P1^ii^	75.48 (5)	P1—O12—Fe3	134.77 (17)
P2—Ca—P1^ii^	97.38 (2)	P1—O12—Fe2	97.02 (14)
P3^vii^—Ca—P1^ii^	104.01 (2)	Fe3—O12—Fe2	128.22 (14)
O11^i^—Ca—P1^v^	77.77 (2)	P1—O13—Fe1	131.13 (12)
O13^ii^—Ca—P1^v^	126.71 (6)	P1—O13—Ca^ix^	109.88 (10)
O23—Ca—P1^v^	124.46 (6)	Fe1—O13—Ca^ix^	118.97 (9)
O23^iv^—Ca—P1^v^	68.54 (6)	O22—P2—O23^iv^	113.70 (10)
O33^v^—Ca—P1^v^	75.48 (5)	O22—P2—O23	113.70 (10)
O33^vi^—Ca—P1^v^	132.14 (5)	O23^iv^—P2—O23	103.06 (16)
P2—Ca—P1^v^	97.38 (2)	O22—P2—O21	107.95 (17)
P3^vii^—Ca—P1^v^	104.01 (2)	O23^iv^—P2—O21	109.13 (10)
P1^ii^—Ca—P1^v^	148.58 (4)	O23—P2—O21	109.13 (10)
O^viii^—Fe1—O^ii^	180.0	O22—P2—Ca	122.57 (13)
O^viii^—Fe1—O13	90.27 (10)	O23^iv^—P2—Ca	51.94 (8)
O^ii^—Fe1—O13	89.73 (10)	O23—P2—Ca	51.94 (8)
O^viii^—Fe1—O13^i^	89.73 (10)	O21—P2—Ca	129.47 (12)
O^ii^—Fe1—O13^i^	90.27 (10)	P2—O21—Fe1	124.75 (8)
O13—Fe1—O13^i^	180.0	P2—O21—Fe1^xiv^	124.75 (8)
O^viii^—Fe1—O21^i^	103.14 (9)	Fe1—O21—Fe1^xiv^	96.97 (11)
O^ii^—Fe1—O21^i^	76.86 (9)	P2—O22—Fe3^xi^	165.2 (2)
O13—Fe1—O21^i^	90.79 (9)	P2—O23—Fe2^ii^	136.88 (12)
O13^i^—Fe1—O21^i^	89.21 (9)	P2—O23—Ca	98.94 (11)
O^viii^—Fe1—O21	76.86 (9)	Fe2^ii^—O23—Ca	124.12 (9)
O^ii^—Fe1—O21	103.14 (9)	O32—P3—O31	109.09 (17)
O13—Fe1—O21	89.21 (9)	O32—P3—O33^xv^	111.49 (11)
O13^i^—Fe1—O21	90.79 (9)	O31—P3—O33^xv^	111.12 (11)
O21^i^—Fe1—O21	180.0	O32—P3—O33^xvi^	111.49 (11)
O—Fe2—O32^ix^	115.92 (13)	O31—P3—O33^xvi^	111.12 (11)
O—Fe2—O23^ix^	96.52 (5)	O33^xv^—P3—O33^xvi^	102.43 (16)
O32^ix^—Fe2—O23^ix^	87.58 (6)	O32—P3—Ca^vii^	122.83 (13)
O—Fe2—O23^x^	96.52 (5)	O31—P3—Ca^vii^	128.08 (12)
O32^ix^—Fe2—O23^x^	87.58 (6)	O33^xv^—P3—Ca^vii^	51.28 (8)
O23^ix^—Fe2—O23^x^	166.93 (11)	O33^xvi^—P3—Ca^vii^	51.28 (8)
O—Fe2—O12	158.95 (11)	P3—O31—Fe3	139.66 (19)
O32^ix^—Fe2—O12	85.13 (12)	P3—O32—Fe2^ii^	139.08 (19)
O23^ix^—Fe2—O12	83.75 (5)	P3^xvi^—O33—Fe3	134.20 (12)
O23^x^—Fe2—O12	83.75 (5)	P3^xvi^—O33—Ca^xiii^	100.10 (10)
O—Fe2—O11	92.67 (12)	Fe3—O33—Ca^xiii^	125.55 (9)
O32^ix^—Fe2—O11	151.41 (11)	Fe2—O—Fe1^xvii^	125.79 (7)
O23^ix^—Fe2—O11	89.23 (6)	Fe2—O—Fe1^ix^	125.79 (7)
O23^x^—Fe2—O11	89.23 (6)	Fe1^xvii^—O—Fe1^ix^	105.66 (12)
O12—Fe2—O11	66.28 (11)		

Symmetry codes: (i) −*x*, −*y*−1, −*z*; (ii) *x*+1, *y*, *z*; (iii) *x*+1, −*y*−1/2, *z*; (iv) *x*, −*y*−1/2, *z*; (v) *x*+1, *y*+1, *z*; (vi) *x*+1, −*y*−3/2, *z*; (vii) −*x*+1, −*y*−1, −*z*+1; (viii) −*x*−1, −*y*−1, −*z*; (ix) *x*−1, *y*, *z*; (x) *x*−1, −*y*−3/2, *z*; (xi) −*x*, −*y*−1, −*z*+1; (xii) *x*, −*y*−3/2, *z*; (xiii) *x*−1, *y*−1, *z*; (xiv) −*x*, *y*+1/2, −*z*; (xv) −*x*, *y*+1/2, −*z*+1; (xvi) −*x*, −*y*−2, −*z*+1; (xvii) −*x*−1, *y*−1/2, −*z*.
